# Familial Thoracic Aortic Aneurysm with Dissection Presenting as Flash Pulmonary Edema in a 26-Year-Old Man

**DOI:** 10.1155/2014/842872

**Published:** 2014-07-07

**Authors:** Sabry Omar, Tyler Moore, Drew Payne, Parastoo Momeni, Zachary Mulkey, Ralph Paone, Kenneth Nugent

**Affiliations:** ^1^Department of Internal Medicine, Texas Tech University Health Sciences Center, 3601 4th Street, Lubbock, TX 79430, USA; ^2^Department of Cardiothoracic Surgery, Texas Tech University Health Sciences Center, 3601 4th Street, Lubbock, TX 79430, USA

## Abstract

We are reporting a case of familial thoracic aortic aneurysm and dissection in a 26-year-old man with no significant past medical history and a family history of dissecting aortic aneurysm in his mother at the age of 40. The patient presented with cough, shortness of breath, and chest pain. Chest X-ray showed bilateral pulmonary infiltrates. CT scan of the chest showed a dissection of the ascending aorta. The patient underwent aortic dissection repair and three months later he returned to our hospital with new complaints of back pain. CT angiography showed a new aortic dissection extending from the left carotid artery through the bifurcation and into the iliac arteries. The patient underwent replacement of the aortic root, ascending aorta, total aortic arch, and aortic valve. The patient recovered well postoperatively. Genetic studies of the patient and his children revealed no mutations in ACTA2, TGFBR1, TGFBR2, TGFB2, MYH11, MYLK, SMAD3, or FBN1. This case report focuses on a patient with familial TAAD and discusses the associated genetic loci and available screening methods. It is important to recognize potential cases of familial TAAD and understand the available screening methods since early diagnosis allows appropriate management of risk factors and treatment when necessary.

## 1. Introduction

Aortic dissection usually occurs in older age groups, but there is a significant proportion of patients with presentations at less than 60 years of age. Thoracic aortic aneurysm and dissection (TAAD) is estimated to occur at a rate of 3 cases per 100,000 individuals per year and is a major cause of death [1]. In the absence of a syndrome associated TAAD, such as Marfan's syndrome, Ehlers-Danlos syndrome, or Loeys-Dietz syndrome, it has been reported that 20% of TAAD cases have a genetic component. These conditions display variable penetrance and severity [2].

## 2. Case Presentation

**Figure 1:**
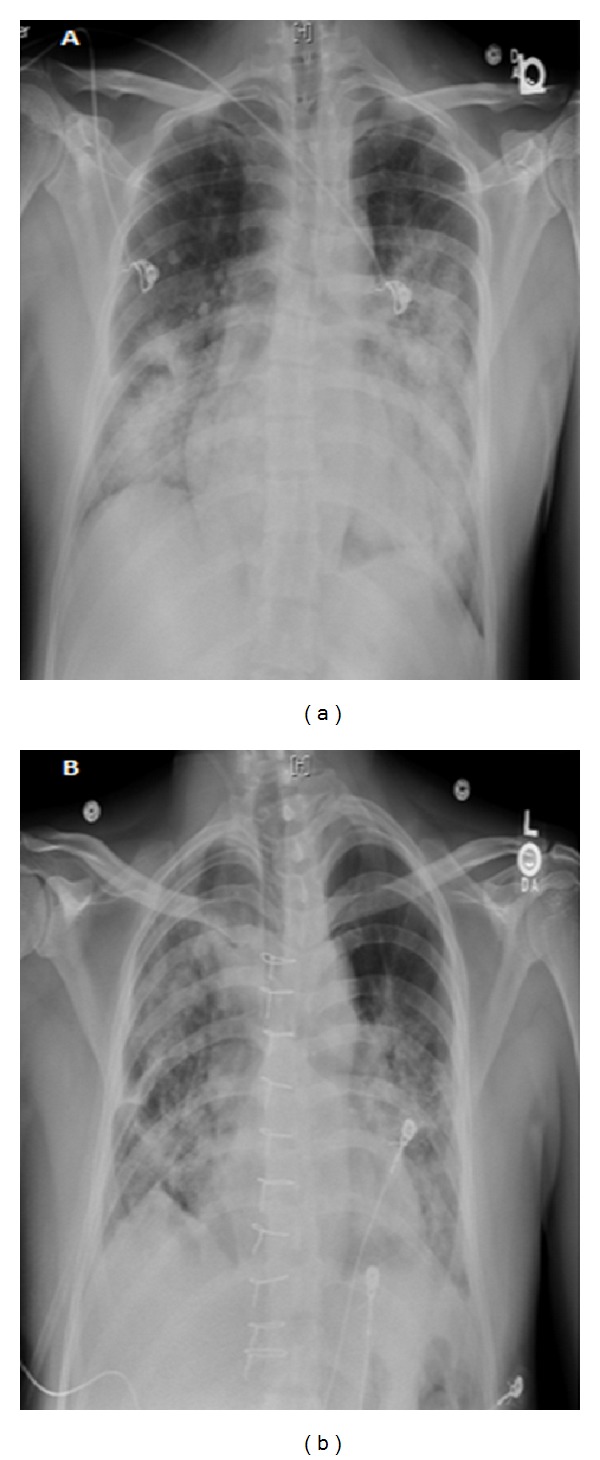
(a) Chest X-ray on admission day, (b) postoperative day 6.

**Figure 2:**
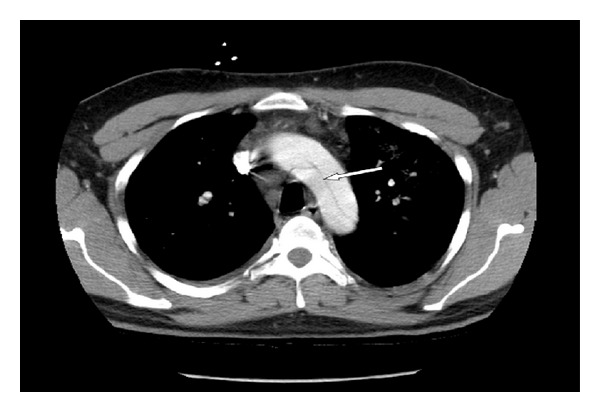
CT scan of chest prior to admission shows aortic dissection.

**Figure 3:**
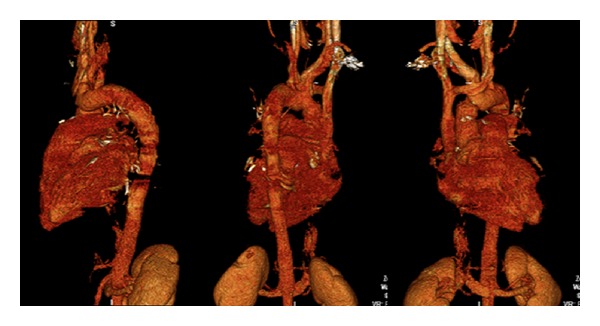
3D reconstruction of the aorta 82 days after aortic repair.

**Figure 4:**
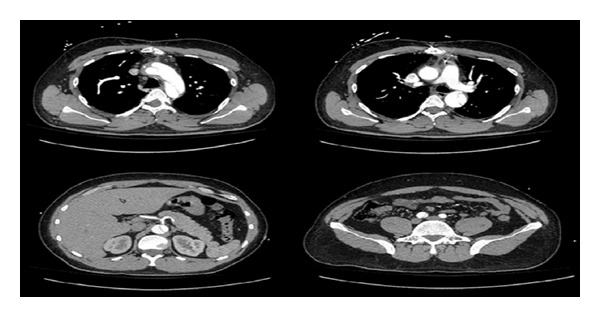
CT scan of second aortic aneurysm and dissection.

**Figure 5:**
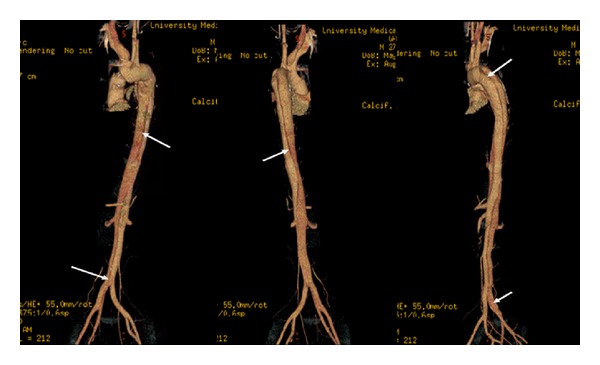
3D reconstruction of the second aortic dissection.

A 26-year-old man had no significant past medical history but had a family history of dissecting aortic aneurysm in his mother at the age of 40. The patient has a normal physical appearance and does not have any features that suggest Marfan's syndrome, Ehlers-Danlos syndrome, Loeys-Dietz syndrome, ANCA-positive vasculitis, or Takayasu's arteritis. The patient does not have disproportionately long extremities, hypertelorism, a bifid or broad uvula, craniosynostosis, cleft palate, club foot, translucent skin, soft velvety skin, easy bleeding, or easy bruising. He presented with cough, shortness of breath, and chest pain for 10 days. The patient's blood pressure on admission was 93/73 mmHg, heart rate was 115 bpm, and respiratory rate was 37 bpm. His laboratory work showed hemoglobin 9 Gm/dL, WBC 16 k/*μ*L, ESR 12 mm/hr, CRP 2 mg/dL, D-dimer 942 ng/mL, BNP 1000 pg/mL, and troponin T 0.05 ng/mL. Chest X-ray at the time of presentation showed bilateral pulmonary infiltrates (Figure 1(a)). He was treated outside the hospital for bronchopneumonia but did not improve. When a CT scan of the chest showed a dissecting aneurysm of the ascending aorta (Figure 2), the patient was transferred to our hospital and successfully underwent aortic dissection repair. Resuspension of the aortic valve and replacement of the ascending aorta with a 24 mm hemashield gold interposition graft were performed. The patient did well postoperatively but remained intubated due to high respiratory rate during CPAP trials. This was likely due to pulmonary edema, as evidenced by bilateral lower lung field opacities seen on chest X-ray (Figure 1(b)). CPAP weaning trials were performed daily, and he was successfully extubated on postoperative day 5. The patient was discharged home on carvedilol 12.5 mg twice a day. A follow-up 3D reconstruction of the aorta 82 days after the surgery is shown in Figure 3. Three months after aortic dissection repair, the patient returned to our hospital with new complaints of sharp back pain. On physical examination, a new diastolic murmur was heard at the left sternal border. CT angiography with 3D reconstruction showed a dissection of the aortic arch with dilated aortic root measuring 5.3 cm. The origins of the innominate artery, left common carotid artery, and left subclavian artery were dissected focally, and the dissection continued down into the common iliac arteries bilaterally (Figures 4 and 5). Echocardiogram showed severe aortic insufficiency with ejection fraction 50%. Because of the progression of his chronic dissection, the dilated aortic root, and severe aortic insufficiency, the patient underwent reoperation. The right axillary artery was cannulated via an 8 mm hemashield graft. Venous drainage was accomplished using a 2-stage venous cannula via the right atrium. Once on bypass, systemic cooling was begun with an eventual bladder temperature of 16–20 degrees C. Cardiac arrest and myocardial protection were accomplished using retrograde cold blood cardioplegia and systemic hypothermia. During the cooling phase, the aortic valve and ascending aorta were replaced with a number 29 On-X valved conduit. The right and left main coronary arteries were reimplanted into the ascending aortic graft. With continuous antegrade cerebral perfusion via the right axillary cannula and a presumed patent circle of Willis, total circulatory arrest was achieved and the aortic arch was replaced. A number 26 hemashield graft was used with a number 12 and number 8 graft attached end to end to the innominate and left carotid arteries, respectively. The proximal ends of the two grafts were attached previously to the arch graft using 5-0 prolene suture. A piece of reversed saphenous vein was used in end to end fashion for the left subclavian artery which was unusually small. The proximal end of the saphenous vein was attached to the arch graft with 5-0 prolene suture. The number 26 hemashield graft was used in an “elephant trunk” fashion. He did well after surgery and was discharged home on warfarin, amlodipine, carvedilol, and losartan. Due to the family history of aortic dissection in his mother at early age, genetic studies on the patient and his children were performed. The genomic DNA was extracted from peripheral blood and was amplified using standard procedures by touchdown PCR of all coding exons with their exon-intron boundaries of ACTA2 and sequencing 6 other genes using forward and reverse primers located in the flanking introns. The PCR products were analyzed by gel electrophoresis and visualized by ethidium bromide staining on 2% agarose gels. The genetic studies revealed no mutations in ACTA2, TGFBR1, TGFBR2, TGFB2, MYH11, MYLK, SMAD3, or FBN1. The patient was referred to John Ritter research program in the University of Texas Medical School at Houston for additional genetic testing.

## 3. Discussion

Thoracic aortic aneurysms and dissections are less common than aneurysms of the abdominal aorta and can affect one or more aortic segments, including the aortic root, ascending aorta, arch, or descending aorta. We describe a male patient with familial TAAD, who does not have any features of the genetic syndromes associated with TAAD. He does have a family history of TAAD in his mother who died at age of 40 secondary to an aortic aneurysm rupture. Syphilitic aortic aneurysm was ruled out with negative syphilis screening. The patient did not have any features that suggest Takayasu's arteritis, such as night sweats, fatigue, arthralgias, weight loss, pulseless arteries in arms or legs, or elevated ESR or CRP. Urine drug screen was negative, ruling out cocaine-induced dissection. Due to the family history of aortic dissection at early age in his mother and the early age of aortic dissection in our patient, the diagnosis of familial thoracic aortic aneurysm with dissection was made. Despite negative genetic studies of the patient for TAAD, we believe that the patient has a gene mutation that has not been identified yet.

TAAD is an uncommon disease that is highly lethal if left untreated. It is associated with degeneration of the aortic media in a process called cystic medial degeneration [3]. Familial TAAD is occasionally associated with brain aneurysms, congenital heart abnormalities, inguinal hernia, scoliosis, or livedo reticularis. Gene mutations identified in TAAD are associated with maintenance of smooth muscle contractile function [4]. Occasionally, mutations in TGFBR1, TGFBR2, or FBN1 are found in families that have increased incidence of TAAD [5]. Multiple loci have been identified, including the TAAD1 locus mapped to 5q13-14, the FAA1 locus mapped to 11q23.3–24, the TAAD2 locus mapped to 3p24-25 with TGFBR2 being the mutant gene, the TAAD3 locus mapped to 15q24–26, and the TAAD4 locus mapped to 10q23-24 with ACTA2 being the mutant gene [6]. There are probably other gene mutations associated with familial TAAD that have not been identified yet. Guo et al. studied genes sequences within the* TAAD5* locus that might be related to smooth muscle cell function, including candidate integrins, actin-binding proteins, and myofibril-related proteins, but did not find disease-causing mutations [7].

Albornoz et al. studied 520 patients with thoracic aortic aneurysm and their families. They reported that an inherited pattern for thoracic aortic aneurysm was present in 21.5% of non-Marfan's syndrome patients. Additionally, 20% of thoracic aortic aneurysms and dissections without known vascular connective tissue syndrome have at least one first degree family member with an arterial aneurysm [8]. In 2006, two large families with autosomal dominant inheritance of TAAD and patent ductus arteriosus were found to have mutations in the gene encoding myosin heavy chain protein 11 (MYH11) on chromosome 16p137 [9]. Subsequently, two additional families with MYH11 mutations were described that showed substantial smooth muscle cell (SMC) disarray and focal hyperplasia of SMCs in the aortic media [10]. Shortly thereafter, the gene encoding SMC *α*-actin* (ACTA2)* on chromosome 10q22–24 was identified in TAAD families who also had additional symptoms, such as livedo reticularis, patent ductus arteriosus, and iris floccule [11]. Recently, loss of function TGFB2 mutations has been reported, which lead to reduced levels of TGF-b2 in smooth muscle cells and fibroblasts explanted from mutation carriers [12].

TAAD is diagnosed by transesophageal echocardiography, CT, MRI, or angiography. Molecular genetic testing of* ACTA2* is a reasonable second step to determine the underlying cause of familial TAAD. Unfortunately, there is no gold standard biomarker to diagnose or to rule out aortic dissection. Suzuki et al. reviewed the development of biomarkers in acute aortic dissection, such as circulating smooth muscle myosin heavy chain, creatine kinase-BB isoenzyme, calponin, CRP, and D-dimer. These biomarkers show marked elevations in patients with acute aortic dissection and may provide temporal profiles similar to the cardiac enzymes used in myocardial ischemia [13]. D-dimer is the most promising biomarker in suspected aortic dissection; it might be helpful as a screening tool to rule out aortic dissection and in risk stratifying patients with suspected aortic dissection. More research is necessary to determine the role of these biomarkers in the diagnosis of aortic dissection.

Medical treatment includes beta adrenergic-blocking agents to reduce hemodynamic stress in individuals with familial TAAD who are at risk of developing aneurysms. Prophylactic surgical repair of the aorta to prevent subsequent dissection or rupture is indicated for individuals with familial TAAD and/or a confirmed mutation in* MYH11* or* ACTA2*, when the diameter of the ascending aorta is between 4.5 and 5.0 cm, and for individuals with familial TAAD when other relatives have experienced aortic dissection with documented minimal enlargement of the aortic diameter [14]. Surgery is indicated in all other patients with TAAD when the ascending aorta or aortic root reaches 5.0 cm, when the rate of dilatation is more than 0.5 cm per year, or when severe aortic stenosis or regurgitation is present [14]. Screening of family members at risk is appropriate if a family-specific mutation is identified. The patient can be offered baseline imaging of the ascending thoracic aorta by echocardiogram, CT, or MRI, as well as molecular genetic testing to clarify their genetic status so that heterozygotes for a pathologic mutation can be monitored appropriately [15].

## 4. Conclusion

Thoracic aortic aneurysms and dissections are a major cause of death associated with many genetic syndromes, including familial TAAD. This case report focuses on a patient with familial TAAD with no associated genetic mutations and discusses the associated genetic loci and available screening methods. Screening begins with testing for the* ACTA2* gene mutation and may involve sequencing 7 other genes associated with familial TAAD. It is important to recognize potential cases of familial TAAD and understand the available screening methods because early diagnosis allows appropriate management of risk factors and treatment when necessary.

## Abbreviations

TAAD: Thoracic aortic aneurysm with dissection
TEE: Transesophageal echocardiography
MRI: Magnetic resonance imaging
CT: Computerized tomography
ACTA2: Smooth muscle aortic alpha-actin.
